# AI-driven designed protein epigenomics

**DOI:** 10.46439/oncology.1.01

**Published:** 2023

**Authors:** Shiri Levy, Hannele Ruohola-Baker

**Affiliations:** 1 Institute for Stem Cell and Regenerative Medicine, University of Washington, School of Medicine, Seattle, WA 98109, USA; 2 Department of Biochemistry, University of Washington, School of Medicine, Seattle, WA 98195, USA

## Abstract

The biological revolutions of computationally designed proteins, induced pluripotent stem cells (iPSCs), and the CRISPR-Cas9 system finally enables modifications that can spur deep understanding of spatial requirement of epigenetic information. This commentary describes the utility of a computationally designed protein, EED Binder (EB), when fused to dCas9 (EBdCas9) for identifying critical sites of PRC2 dependent histone H3K27me3 marks in the chromatin. By using EBdCas9 and gRNA, PRC2 function can be inhibited at specific loci, resulting in precise reduction of EZH2 and H3K27me3 marks, and in some (but not all) locations, activation of the gene and functional outcomes (such as regulation of cell cycle or trophoblast transdifferentiation). Interestingly, a functional TATA box located more than 500bp upstream of a TBX18 TSS was found to be repressed by PRC2, supporting the theory that epigenetic regulators control the repression of transcriptional elements on the promoter region. The EBdCas9 technology may provide a useful tool for controlling gene regulation through epigenomic control.

While H3K27me3 marks are broadly decorating the promoter regions of developmental genes, and generally associate with gene repression [1], a central question in the field is which specific PRC2-dependent H3K27me3 marks (if any) are essential for gene repression. The development of a new tool [2,3], for the first time enables spatial dissection of specific H3K27me3 histone marks associated with individual genes.

PRC2 (Polycomb Repressive Complex 2) plays a crucial role in the process of determining the fate of cells during development by modifying chromatin through a specific histone modification called H3K27me3. Among the PRC2 proteins which facilitate new H3K27me3 modification on the chromatin are the reader EED which anchor the PRC2 complex to the chromatin and the writer/catalyzer, methyltransferase, EZH2 which methylates the lysine 27 sidechain in histone 3 (H3K27). To understand the requirement of the PRC2 in different developmental stages of pluripotency we generated a computer-designed protein that binds the EZH2 binding site in EED (EED binder; EB), thereby competing with EZH2 [2]. EB binding to EED inhibits EZH2 interactions with EED, resulting in EZH2 and JARID2 degradation, a global reduction of H3K27me3 marks, and ultimately, activation of thousands of PRC2-dependent developmental genes [2]. To answer the question of whether any specific H3K27me3 marked nucleosomes are critical alone for the control of gene expression we fused EB to catalytically dead Cas9 (EBdCas9). In the past decade, numerous groups utilized dCas9 as a versatile tool for epigenomic editing using effector and repressor molecules to shed light on gene regulation, cellular differentiation, and as potential therapeutic agents in pathological diseases and regenerative medicine [4–19]. However, EBdCas9 is the first computer designed protein used in this context.

Targeting EBdCas9 and appropriate single guide RNA to specific chromatin locus on a promoter region of a gene, such as the transcription factor in pacemaker cells, TBX18 results in PRC2 repression and activation of TBX18 gene expression. Moreover, this derepression of TBX18 gene was inherited to the daughter cells since Tbx18 was expressed in subsequent cell divisions after removal of EBdCas9 and the guide RNA. While the repression of EZH2, H3K27me3, and JARID2 by EBdCas9/gRNA were initially limited to a single gRNA determined locus, in specific locations epigenomic changes readily spread onto the promoter region towards the transcription start site, TSS. Additionally, the previously facultative heterochromatin was replaced with histone marks associated with gene activation, such as H3K27ac. The enzyme which catalyzes the transfer of acetyl marks to histone, histone acetyltransferase, p300 was also recruited to the locus. The generated euchromatin was characterized by the presence of RNA polymerase 2, and the hydroxylation of DNA methyl marks at CpG island proximal to TSS. Therefore, the targeted PRC2 inhibition results in epigenomic remodeling, native activation of the transcriptional machinery, gene expression and inherited epigenomic memory.

Interestingly, we identified two kinds of PRC2 responsive loci, the loci in which PRC2 function was essential for gene repression, and loci in which PRC2 did not function to repress transcription. We sought to understand what is/are the molecular mechanism/s responsible for such profoundly different effects.

In the case of TBX18, one unique identifier was detected on the promoter region of the gene, in close proximity to the impactful single guide RNA that resulted in substantial epigenomic and transcriptional changes. The Element prediction tool [20] identified a potential TATA box in close proximity to the guide RNA. However, this domain was atypically far, >500bp upstream of TSS (typically TATAbox is found ~30bp from TSS). Deletion of this identified upstream TATA box eliminated the epigenomic remodeling and transcriptional activation of the TBX18 proving that the domain was a bona fide TATA box. Moreover, targeted PRC2 inhibition by EBdCas9 allowed the recruitment of TATA Binding Protein (TBP) proving that the distal TATA box normally masked by PRC2 repressive marks can be functional in regulating TBX18 expression.

Control of epigenomic regulation by AI-designed proteins holds immense therapeutic promise for human disease without using chemical drugs or manipulation of endogenous gene sequence. Diffuse Midline Glioma (DMG), is an aggressive form of brain cancer that develops in glial cells in the pons, primarily affecting children [21,22]. A lysine-to-methionine mutation in oncohistone H3.3 gene (H3.3K27M) defines DMG tumors [21,22]. In rapidly dividing cells, such as in DMG, the cell cycle regulator *p16* is repressed due to hypermethylation at the promoter area, however, the capacity to activate p16 for cell cycle arrest could lead to a breakthrough in DMG therapeutics. Transient introduction of EBdCas9 and appropriate guide RNA to p16 gene promoter region of DMG patient cell line led to reduction of EZH2 and H3K27me3 epigenomic marks, p16 transcript and protein activation. Importantly, impactful reduction of over 50% in DMG cell replication and viability was observed in these experiments [3], supporting the validity of AI-designed EpiBinders in future therapies.

Finally, regenerative medicine offers the potential to repair diseased cells and innovatively provide new gene and cell therapies for previously intractable conditions. One of the hallmarks of regenerative medicine is the ability to transdifferentiate one cell type to another beyond their natural bifurcation point. Likewise, the extraembryonic placental vs embryonic cellular fate decision (the first in eutherian development) is dependent on PRC2 [23]. While abundant H3K27me3 marks are associated with embryonic lineage, genome-wide depletion of H3K27me3 marks is associated with extraembryonic placental lineage [23–28]. Primed stage iPSCs are considered embryonic-like cells, which have already passed the bifurcation point and cannot readily generate extraembryonic lineage. Therefore, we used primed stage iPSC as a transdifferentiation model to directly reprogram primed iPSC to extraembryonic placental cells. Directing EBdCas9 with appropriate guide RNAs to multiple transcription factor genes at primed iPSC stage resulted in the transdifferentiation of iPSCs to placental progenitor cells (confirmed by RNAseq analysis, unique placental morphological changes, and placental markers).

## Conclusions

The field of regenerative medicine is rapidly growing and offers a wide range of potential therapies for the treatment of various diseases. One promising area of research within regenerative medicine is the use of epigenetic modifiers towards epigenetic therapies. The absolute gem in epigenetic therapies is the ability to correct defects in gene regulation without altering the underlying genetic code. In this commentary we have discussed how a computer designed mini-protein fused to dCas9 can specifically activate genes through releasing epigenetic repression [3]. In the future these novel AI-designed epigenetic competitor- or recruiter-proteins may join the forefront of the epigenetic modifiers towards effective and impactful therapeutics. Already a generation of AI-designed EpiBinder proteins that aim to target different epigenomic pathways is underway, suggesting a birth of an emerging revolution; AI-driven designed protein epigenomics.

## Figures and Tables

**Figure 1.**
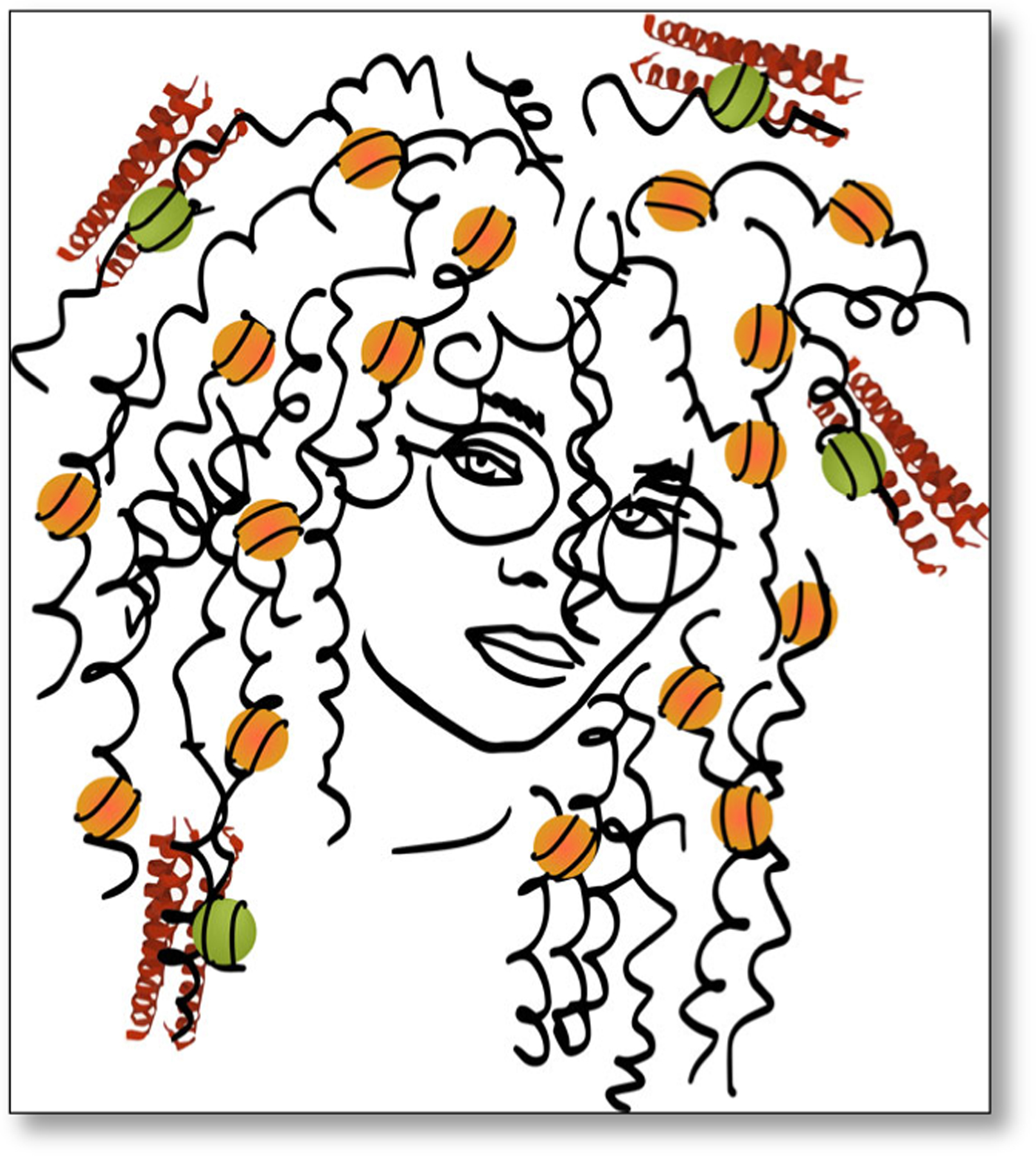
Lady Histone’s curly hair mimics heterochromatin (orange rolls) regions, and euchromatin (green rolls) regions following epigenomic remodeling using the novel designed protein that inhibits the repressive epigenetic modifications.
